# Exploring the resilience of wheat crops grown in short rotations through minimising the build-up of an important soil-borne fungal pathogen

**DOI:** 10.1038/s41598-018-25511-8

**Published:** 2018-06-22

**Authors:** V. E. McMillan, G. Canning, J. Moughan, R. P. White, R. J. Gutteridge, K. E. Hammond-Kosack

**Affiliations:** 1 Department of Biointeractions and Crop Protection, Rothamsted Research, Harpenden, Hertfordshire, AL5 2JQ UK; 2 Department of Computational and Analytical Sciences, Rothamsted Research, Harpenden, Hertfordshire, AL5 2JQ UK

## Abstract

Given the increasing demand for wheat which is forecast, cropping of wheat in short rotations will likely remain a common practice. However, in temperate wheat growing regions the soil-borne fungal pathogen *Gaeumannomyces tritici* becomes a major constraint on productivity. In cultivar rotation field experiments on the Rothamsted Farm (Hertfordshire, UK) we demonstrated a substantial reduction in take-all disease and grain yield increases of up to 2.4 tonnes/ha when a low take-all inoculum building wheat cultivar was grown in the first year of wheat cropping. Phenotyping of 71 modern elite wheat cultivars for the take-all inoculum build-up trait across six diverse trial sites identified a few cultivars which exhibited a consistent lowering of take-all inoculum build-up. However, there was also evidence of a significant interaction effect between trial site and cultivar when a pooled Residual Maximum Likelihood (REML) procedure was conducted. There was no evidence of an unusual rooting phenotype associated with take-all inoculum build-up in two independent field experiments and a sand column experiment. Together our results highlight the complex interactions between wheat genotype, environmental conditions and take-all inoculum build-up. Further work is required to determine the underlying genetic and mechanistic basis of this important phenomenon.

## Introduction

Plant pathogens are one of the greatest threats to global food security^1–3^. Due to economic and technological factors as well as the increasing demand for food there has been a trend to grow many of our major food crops in shorter rotations^4,5^. However, under these conditions there is an increased risk of the build-up of soil-borne plant pathogens and pests that can limit the yields attained^6–11^. Typically soil-borne diseases have been hard to control due to their broad host ranges, difficulties of targeting the pathogen population in the soil with chemistry and the slow progress when breeding for genetic resistance to root diseases^12–15^.

The demand for wheat (*Triticum aestivum*) is expected to increase by 60% by 2050 (www.wheatinitiative.org). To meet this demand and protect wheat yield potential it will be essential to effectively control soil-borne pests and pathogens. Worldwide it has been estimated that 40% of wheat crops are not preceded by an effective break crop, forage or fallow^16^. In temperate wheat producing regions the soil-borne fungus *Gaeumannomyces tritici* can then become a major constraint on wheat productivity^17,18^. Fungal inoculum builds up in the soil during the first year of wheat cropping and can then cause severe root disease if a second wheat crop is grown on the same site. The fungus is a necrotrophic pathogen with a relatively broad host range, being able to infect various grass species including the cereal species barley, triticale and rye^19,20^. Moderate and severe take-all infections can result in grain yield losses and poor grain quality. In addition substantial amounts of unused nitrogen fertiliser, mostly in the form of nitrates, can be left behind in the soil due to poor root function^21^. In NW Europe the influence of climate change has been predicted to lead to even greater yield losses due to take-all in the future^22^.

Genetic resistance to take-all disease remains elusive. Modern hexaploid wheat cultivars are considered fully susceptible to take-all disease, although more distantly related cereal and grass species can show moderate to strong resistance phenotypes^17,23–25^. However, these sources of resistance have still to be successfully transferred into hexaploid wheat. Despite the high susceptibility of hexaploid wheat to take-all disease, we have previously reported on the identification of a novel genetic trait called take-all inoculum build-up (TAB) within semi-modern wheat cultivars when grown as a first wheat crop in the rotation^26^. Low TAB cultivars were able to minimise take-all inoculum build-up in the soil as measured using a soil infectivity bioassay taken post-harvest.

In our present study, the first hypothesis to test was whether post-harvest differences in inoculum build-up could be maintained through the inter-crop period to influence the resulting incidence and severity of take-all disease and grain yield in a following second wheat crop in the rotation. Previous research suggests that there can be considerable change in *G*. *tritici* inoculum levels during the inter-crop period related to the environmental conditions, management strategy and length of time before sowing the following crop^27–32^. To have any impact on disease the differences in inoculum build-up between cultivars that we have previously reported need to be maintained until the sowing of the second crop. It is the amount of viable inoculum in the soil at the time of sowing a susceptible crop that influences the initial primary infection rate in the autumn and the resulting final disease incidence and severity of that crop^33^. In this study we conducted wheat cultivar rotation field trials over multiple field seasons to test and quantify the effect of this new 1^st^ wheat genetic trait on second wheat crop health and performance. An added component of these experiments was the inclusion of a range of different commercial elite wheat cultivars in the second wheat position to test whether particular combinations of first and second wheat cultivars work additively, synergistically or antagonistically to maximise or minimise take-all disease severity.

Secondly, we aimed to evaluate the potential practical use of the TAB trait in disease management strategies by testing how robustly the TAB trait was expressed in modern elite wheat cultivars, grown as a first wheat in the rotation, under a range of environmental and agronomic conditions typical of standard wheat cultivation practices in the UK.

Finally, we tested the hypothesis that differences in root number, biomass or deep rooting existed between cultivars with contrasting TAB phenotypes. The mechanism of differential build-up is currently unknown but we postulated that cultivars with higher root numbers, increased root biomass or shallower rooting could potentially exacerbate the build-up of *G*. *tritici* inoculum. We examined rooting traits in two independent 1^st^ wheat field experiments in different field seasons on the Rothamsted Farm (Hertfordshire, UK) and in a controlled environment sand column wax layer experiment.

## Results

### Wheat cultivar rotation field experiments

To explore the impact of the 1^st^ wheat take-all inoculum build-up trait on take-all root disease severity and grain yield in the following second wheat crop, four rotation field trials were done over the period 2009 to 2013 in Hertfordshire, UK. In year 1 large strips of the low TAB cultivar Cadenza or the high TAB cultivar Hereward were grown followed in year 2 by one of eight UK commercial cultivars. As expected in each trial, the take-all inoculum build-up in the 1^st^ year was dependent on the wheat cultivar grown (Table 1). Overall take-all inoculum levels were high in 2011 and 2012 (average across trial site 2011 = 28.6% roots infected in soil core bioassay, 2012 = 27.3%), moderate in 2009 (21.6%) but extremely low in 2010 (<6%). Only under the extremely low build-up conditions in 2010 was no statistically significant effect of first wheat cultivar on take-all build-up observed (Table 1). Previous studies have generally found that hot and dry weather in the spring and summer are unfavourable for the development of take-all disease^18,34^. The very low build-up in 2010 is therefore presumed to be a result of the low rainfall and higher temperatures in June and July 2010 compared to the other seasons (Supplementary Table S1).

**Table 1:** Take-all infectivity of the soil after harvest of the source cultivars (Cadenza and Hereward) grown as a first wheat crop in the rotation.

	Post-harvest soil core bioassay			
	Logit % roots infected with take-all (back-transformed means)			
Source cultivar	2009/R/CS/688^1^	2010/R/CS/706^2^	2011/R/CS/719	2012/R/CS/725
Cadenza	−1.01 (11.1)	−1.87 (1.8)	−0.73 (18.4)	−0.94 (12.8)
Hereward	−0.67 (20.1)	−1.72 (2.6)	−0.31 (34.7)	−0.24 (37.6)
d.f.	3	3	3	3
SED (logits)	0.084	0.173	0.115	0.150
F Probability	0.027	0.450	0.034	0.019

^1^Rothamsted field trial codes.

^2^Dry weather from April-July 2010 restricted take-all build-up.

**Figure 1:**
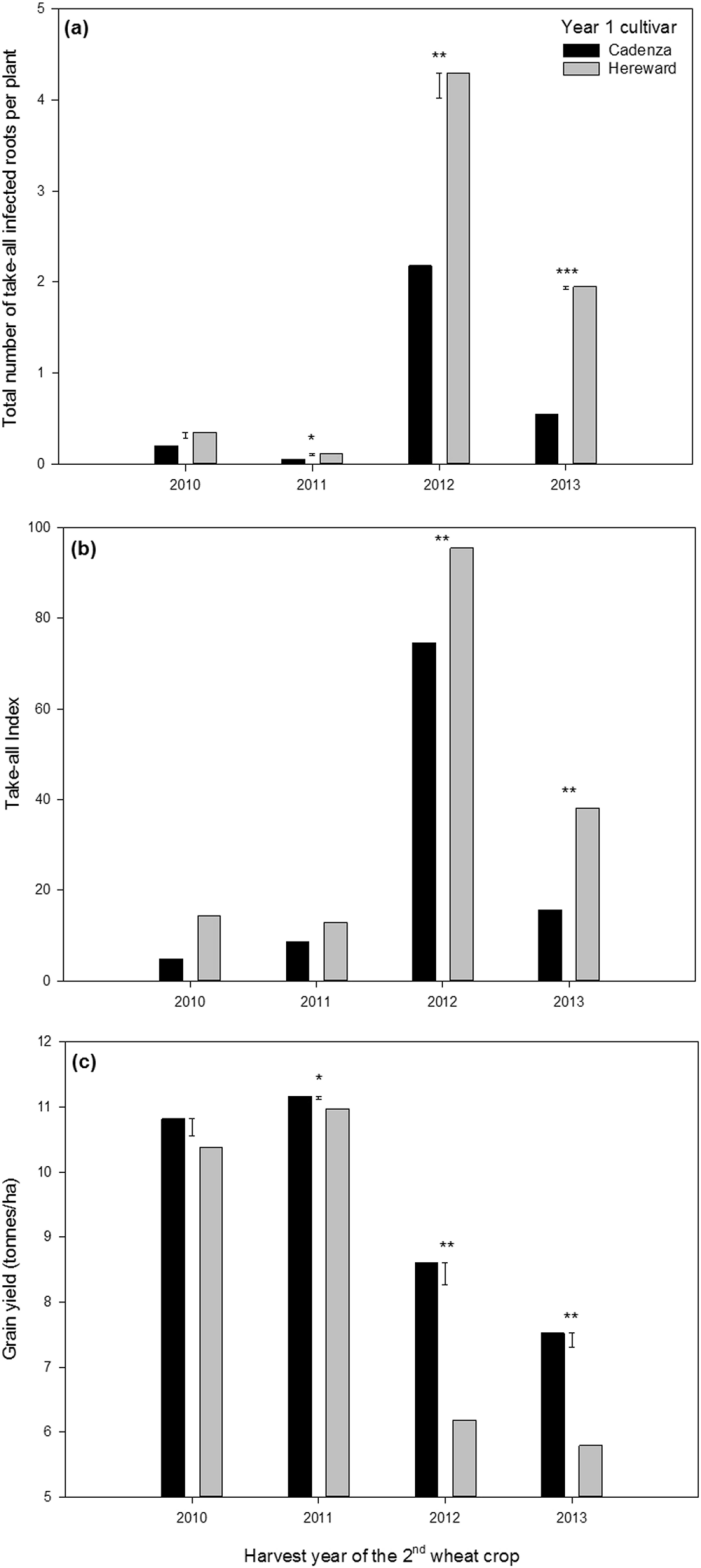
Mean take-all disease severity on the roots in the spring (**a**) and summer (**b**) and grain yield (**c**) of the eight winter wheat cultivars sown as a second wheat crop after the low TAB cultivar Cadenza and high TAB cultivar Hereward were sown in the first year of wheat cropping. Error bars in panels (a) and (c) show the standard error of the difference between the means for comparisons in each year. In panel (b) the back-transformed means on the take-all index scale are shown (Logit of the take-all index: 2010, Cadenza = −1.432, Hereward = −0.873, SED = 0.183; 2011, Cadenza = −1.151, Hereward = −0.931, SED = 0.094; 2012, Cadenza = 0.550, Hereward = 1.578, SED = 0.114; 2013, Cadenza = −0.823, Hereward = −0.232, SED = 0.054). *Significantly different from zero at the 0.05 level of probability, **Significantly different from zero at the 0.01 level of probability, ***Significantly different from zero at the 0.001 level of probability.

To explore the combined effects of wheat genetics and environmentally induced alterations to take-all inoculum levels in the soil on second wheat performance, take-all root disease levels as well as grain and straw yield of the second wheat crop were determined using eight distinct UK wheat cultivars selected from the four nabim (National Association of British and Irish Millers) end use quality categories. These were the group 1 wheats with consistent bread making quality Gallant, Hereward, Solstice and Xi19, group 2 wheats with bread-making potential Cordiale and Einstein, group 3 biscuit making wheat Robigus and group 4 feed wheat Duxford. Irrespective of cultivar grown in year 2 there was strong evidence for the occurrence of less severe take-all root disease in both the spring and summer where the low TAB cultivar Cadenza had been sown in the first year of wheat cropping. (Fig. 1a,b). This translated to a significantly higher second wheat grain yield in three out of the four experiments (Fig. 1c). This grain yield difference ranged from 0.2 tonnes/ha in 2011, when take-all disease pressure coming from the 2010 crop was very low, to nearly 2.5 tonnes/ha in 2012 when take-all disease was most severe.

**Figure 2:**
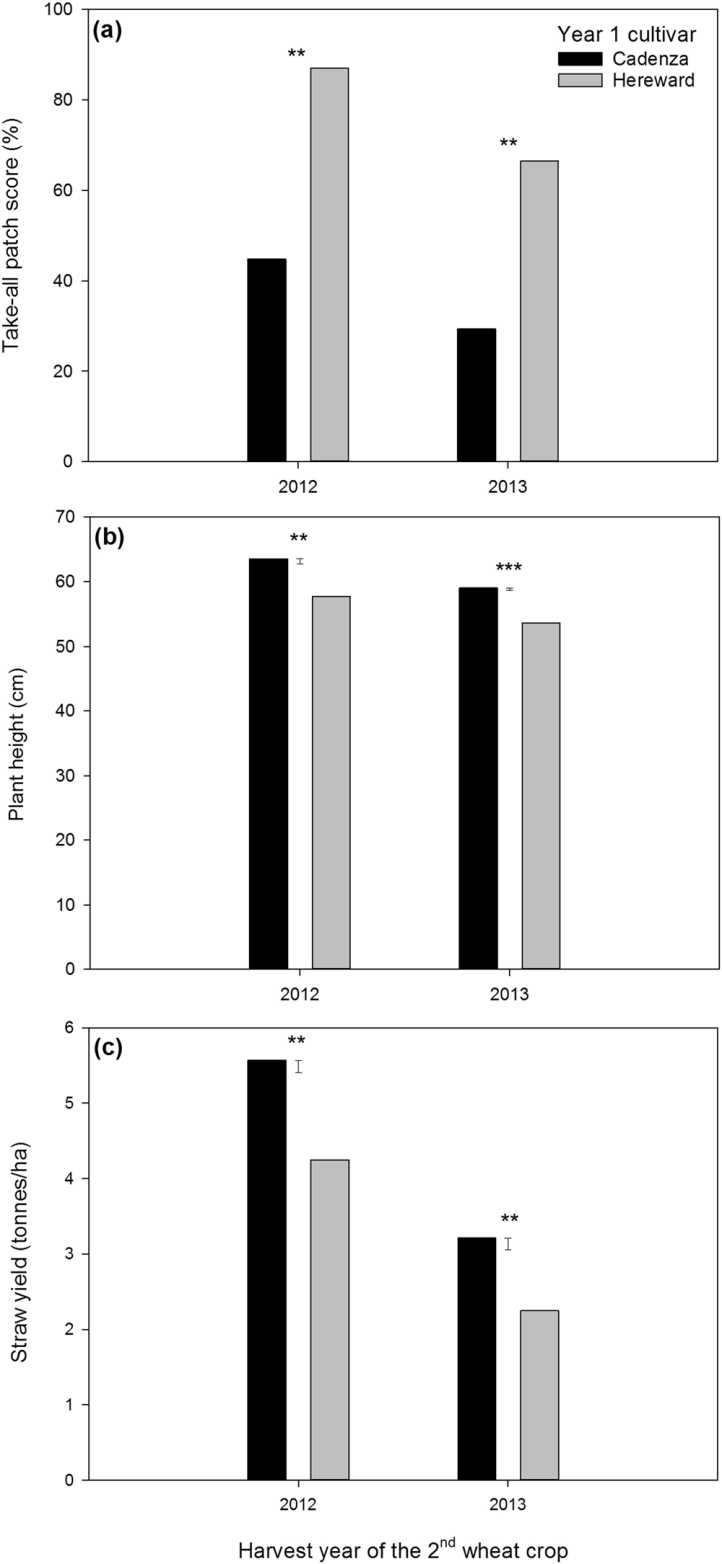
Take-all patch score (**a**), plant height (**b**) and straw yield (**c**) of eight winter wheat cultivars sown as a second wheat crop after the low TAB cultivar Cadenza and high TAB cultivar Hereward were sown in the first year of wheat cropping. In panel (a) the back-transformed means on the percentage scale are shown (Logit take-all patch score: 2012, Cadenza = −0.103, Hereward = 0.939, SED = 0.141; 2013, Cadenza = −0.427, Hereward = 0.352, SED = 0.112). Error bars in panels (b) and (c) show the standard error of the difference between the means for comparisons in each year. *Significantly different from zero at the 0.05 level of probability, **Significantly different from zero at the 0.01 level of probability, ***Significantly different from zero at the 0.001 level of probability.

Visible above-ground symptoms of take-all developed across the trial sites as characteristic patches of stunted, yellowing and prematurely ripening wheat plants in the second wheat crops in 2012 and 2013. In these years additional above-ground measurements were taken to quantify this effect: take-all patch score, plant height and straw yield. The take-all patch score of the second wheat cultivars was reduced where the low TAB cultivar Cadenza had been grown in year 1 compared to high TAB Hereward (Fig. 2a). This was also reflected by the significantly taller plant height and increased straw yield in the second wheats after Cadenza compared to Hereward (Fig. 2b,c).

**Figure 3:**
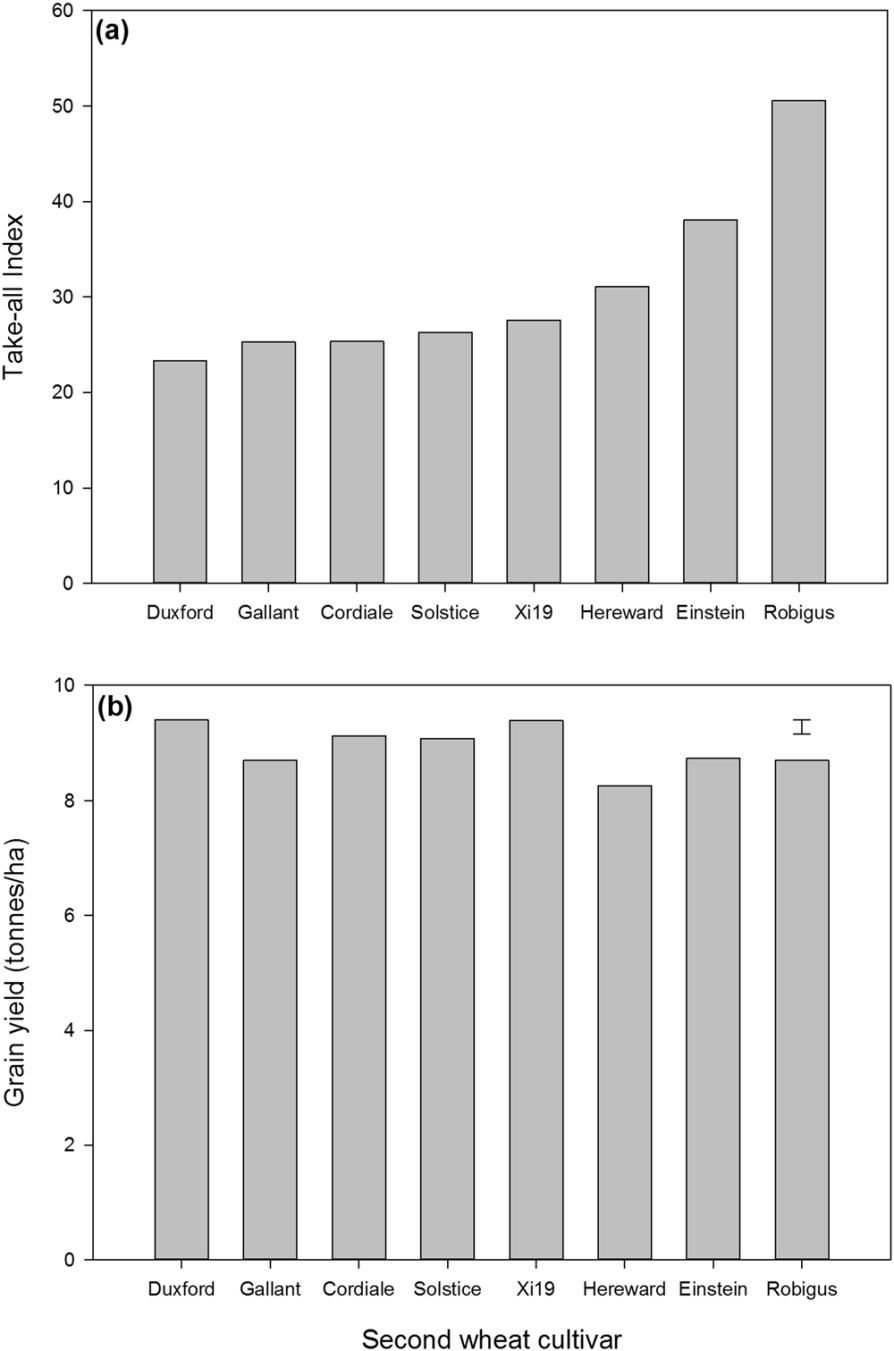
Take-all severity (**a**) and grain yield (**b**) of eight winter wheat cultivars sown as a second wheat crop in the rotation in a combined analysis of the four rotational field experiments (Logit of the take-all index F Pr < 0.001; Grain yield, F Pr < 0.001). In panel (a) the back-transformed means on the take-all index scale are shown (Logit of the take-all index; Duxford = −0.596, Gallant = −0.542, Cordiale = −0.540, Solstice = −0.515, Xi19 = −0.482, Hereward = −0.399, Einstein = −0.243, Robigus = 0.011, SED = 0.106). The error bar in panel (b) shows the standard error of the difference between the means.

In all four rotation field experiments there were no significant interaction effects detected between year 1 source cultivar and year 2 cultivar for any disease or yield variable. However, there was evidence of an independent main effect of second wheat cultivar (P < 0.001) on the severity of take-all disease with Robigus being statistically more severely infected than all of the other cultivars (Fig. 3a). There was also a significant effect of second wheat cultivar on yield (Fig. 3b, P < 0.001) but the relationship of this with take-all disease severity is unclear because of presumed inherent differences between cultivars in yield per se and potential differences between cultivars in their tolerance of take-all disease.

### AHDB winter wheat Recommended List TAB phenotyping

In 2013/14 and 2014/15, to explore the robustness of the TAB trait across varying environmental and agronomic conditions, seventy-one AHDB Recommended or candidate commercial wheat cultivars were phenotyped using the soil core bioassay method at six AHDB Recommended List field experimental sites (8 cultivars represented at 1 site only, 7 cultivars at 2 sites, 18 cultivars at 3 sites, 4 cultivars at 4 sites, 15 cultivars at 5 sites and 19 cultivars at all six sites). The sites differed in their geographic location, soil type, prior break crop and sowing date (Supplementary Table S2).

In 2013/14 a large range in the mean cultivar take-all soil infectivity, measured as the percentage roots infected with take-all, was detected at each trial site analysed individually (Trial 1 Hampshire Sutton Scotney = 16.8–68.5%, Trial 2 Hampshire Broughton = 9.2–47.7%, Trial 3 Oxfordshire Alkerton = 14.2–69.2%). At two of the three sites in 2013/14 there was a close to significant or significant effect of cultivar on take-all inoculum build-up during the first wheat crop (Trial 1, % plants infected with take-all P < 0.001, % roots infected with take-all P = 0.054; Trial 3, % plants infected with take-all P = 0.033, % roots infected with take-all P = 0.006) (Supplementary Table S6a).

In 2014/15 the overall amount of take-all build-up, measured as the percentage roots infected with take-all in the soil core bioassay, was lower than in 2013/14 (Trial 4 Hampshire Broughton = 0.0–25.2%, Trial 5 Kent Wye = 0.2–29.9%, Trial 6 Oxfordshire Alkerton = 3.6–36.9%). Under this lower inoculum build-up situation and higher residual variability there was no significant effect of cultivar on TAB at any of the sites with the exception of the percentage plants infected with take-all for Trial 6 (P = 0.049) (Supplementary Table S6a).

At all of the trial sites the mean cultivar percentage plants and roots infected with take-all in the soil core bioassay was not correlated with 1^st^ wheat yield performance (Supplementary Table S3).

In the pooled REML analysis of all six sites for the percentage of roots infected with take-all there was a significant interaction between year and trial site detected (P < 0.001) (Supplementary Table S4). In 2013/2014 all three sites had a similar level of *G*. *tritici* inoculum build-up. In contrast in 2014/2015 the Oxfordshire Alkerton trial site (Trial 6) had a higher average infectivity than the Hampshire or Kent sites in that year. The Oxfordshire trial site was drilled on the same date as the Hampshire trial site in 2014/2015 but after different break crops (winter beans vs grain peas) and on different soil types (light sandy soil vs medium soil with some chalk) (Supplementary Table S2). However, no difference between trial sites in these regions with the same prior break crops and soil types but different sowing dates was identified the previous year in 2013/2014.

In the pooled analysis there was a highly significant main effect of cultivar on take-all infectivity measured as the percentage roots infected with take-all (P < 0.001) and no significant difference in the response of cultivars across the years and trial sites (Year.Trial.Cultivar P = 0.058, Table 2, Supplementary Table S6b), regardless of the different environmental and agronomic variables and the overall conduciveness of the sites to take-all build-up. In total eight cultivars had less than 10% infected roots in the pooled REML analysis (Table 2). Three of these cultivars had been tested across 5 of the 6 trials sites, namely Grafton, KWS Cashel and Cordiale. In contrast twelve cultivars had more than double the percentage infected roots (20% or more) in the pooled analysis with six cultivars, KWS Croft, Revelation, Costello, Zulu, KWS Lili and KWS Santiago tested across both field seasons in either 5 or all 6 of the trial sites.

**Table 2:** Pooled TAB phenotyping analysis of the percentage roots infected with take-all at the six sampled AHDB experimental sites in 2013/14 and 2014/15.

Cultivar name	Logit % roots infected with take-all (Bt^1^ mean)		Number of sites	Cultivar name	Logit % roots infected with take-all (Bt^1^ mean)		Number of sites
SY-111643	−1.501	(4.73)	1	Butler	−0.853	(15.37)	3
Belgrade	−1.464	(5.08)	3	Alchemy	−0.826	(16.08)	4
KWS Siskin	−1.333	(6.50)	3	RGT Adventure	−0.825	(16.12)	3
Grafton	−1.332	(6.52)	5	Britannia	−0.823	(16.17)	5
Amplify	−1.244	(7.67)	3	Icon	−0.818	(16.31)	4
RGT Marlborough	−1.163	(8.89)	2	Sherlock	−0.816	(16.36)	3
KWS Cashel	−1.117	(9.68)	5	Horatio	−0.815	(16.40)	6
Cordiale	−1.111	(9.77)	5	Claire	−0.808	(16.58)	5
Graham	−1.098	(10.02)	3	Panacea	−0.807	(16.61)	2
Crusoe	−1.087	(10.21)	5	Ruskin	−0.803	(16.71)	3
Gallant	−1.079	(10.35)	6	RGT Illustrious	−0.793	(17.00)	3
RGT Pembroke	−1.066	(10.61)	3	Twister	−0.791	(17.04)	4
Lancaster	−1.043	(11.06)	1	Cougar	−0.790	(17.07)	6
Panorama	−1.028	(11.35)	2	Chilton	−0.784	(17.26)	3
Relay	−1.013	(11.65)	6	Evolution	−0.784	(17.26)	5
KWS Gator	−0.998	(11.96)	5	Scout	−0.766	(17.77)	5
Cubanita	−0.993	(12.08)	6	KWS Silverstone	−0.763	(17.86)	3
Invicta	−0.992	(12.09)	6	RGT Scrummage	−0.752	(18.18)	2
KWS Crispin	−0.976	(12.43)	3	Skyfall	−0.752	(18.18)	5
KWS Trinity	−0.956	(12.89)	6	KWS Kielder	−0.733	(18.77)	6
Jorvik	−0.945	(13.12)	2	Tuxedo	−0.729	(18.87)	1
KWS Barrel	−0.945	(13.13)	3	KWS Leon	−0.718	(19.21)	1
Reflection	−0.942	(13.20)	6	Myriad	−0.715	(19.30)	4
Spyder	−0.941	(13.22)	3	KWS Croft	−0.686	(20.25)	5
Leeds	−0.931	(13.45)	6	Revelation	−0.678	(20.48)	6
Monterey	−0.929	(13.50)	5	Costello	−0.677	(20.52)	6
KWS Tempo	−0.926	(13.57)	3	Conqueror	−0.660	(21.08)	2
Beluga	−0.912	(13.89)	2	Zulu	−0.658	(21.14)	5
Solstice	−0.910	(13.94)	6	KWS Lili	−0.653	(21.31)	6
Dickens	−0.899	(14.20)	6	KWS Basset	−0.647	(21.52)	3
Delphi	−0.899	(14.21)	6	KWS Santiago	−0.597	(23.27)	6
JB Diego	−0.878	(14.72)	6	SY-111539	−0.504	(26.72)	1
Energise	−0.875	(14.80)	6	SY-111570	−0.462	(28.41)	1
Mosaic	−0.870	(14.94)	3	LGW64	−0.325	(34.32)	1
Viscount	−0.856	(15.28)	5	Icebreaker	−0.290	(35.89)	1
RGT Conversion	−0.856	(15.30)	5				
SED	0.211						
F Probability	<0.001						

^1^Bt = Back-transformed.

**Figure 4:**
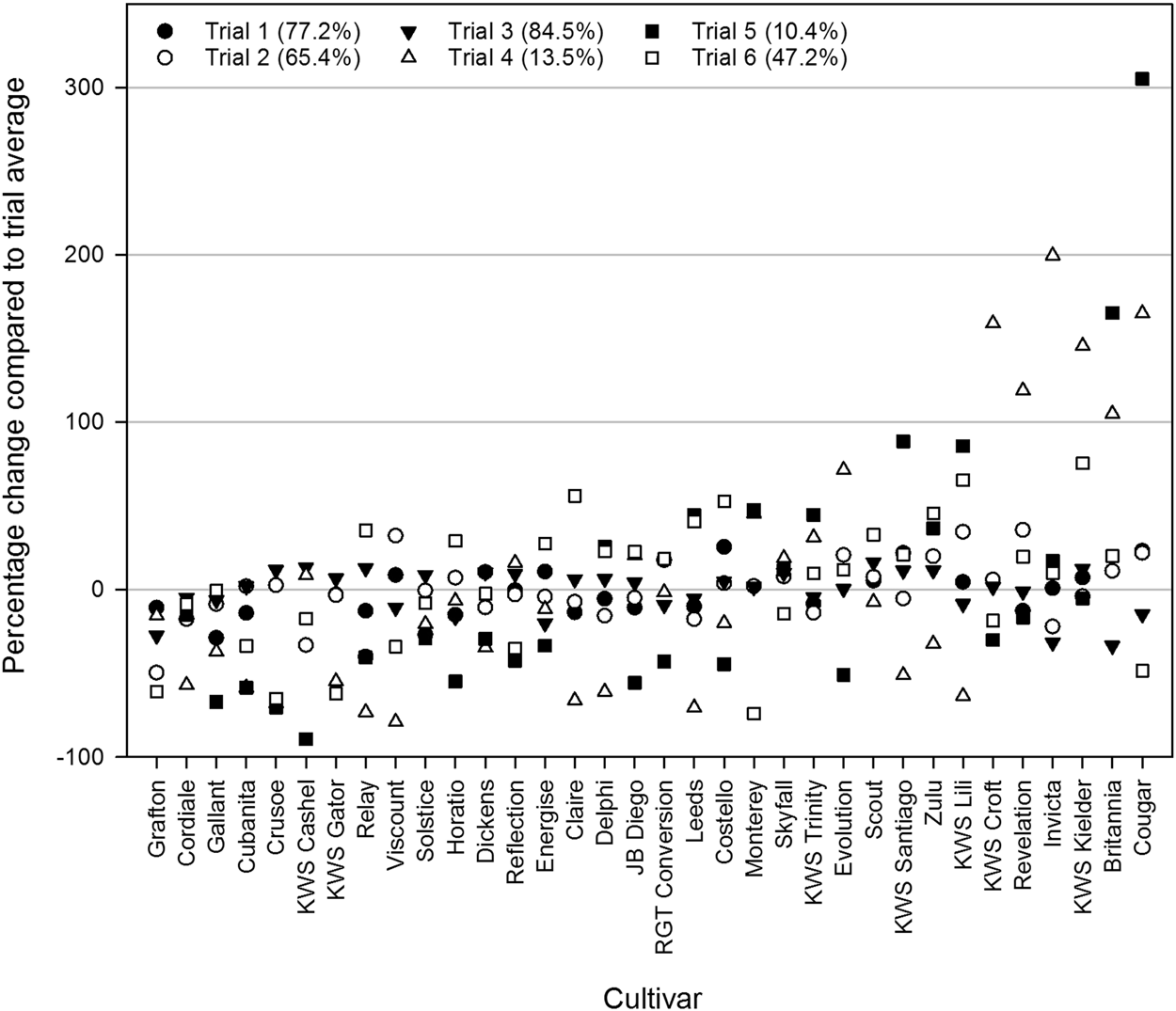
Relative change per cultivar in the percentage plants infected in the soil core bioassay compared to the trial site average. Legend shows the trial site and in brackets the average percentage plants infected with take-all at each site.

In contrast to the percentage roots infected with take-all there was a statistically significant interaction effect for the percentage of plants infected with take-all across the years and trial sites (Year.Trial.Cultivar P < 0.001, Supplementary Table S6b), indicating that the cultivars show different patterns of response across the trials. Overall the percentage of plants and percentage of roots infected with take-all in the soil core bioassay at each site were highly correlated (Rs = 0.78–0.97, P < 0.001) but this difference in interaction effect in the pooled analysis suggests that the percentage of plants infected with take-all in the soil core bioassay shows a more consistent difference in the pattern of response of the cultivars across the trial sites. For the cultivars which were represented at either 5 or 6 trials sites (34 cultivars) their percentage increase or decrease in take-all build-up relative to the overall trial average of percentage plants infected is shown in Fig. 4. Cordiale, Gallant and Grafton show a consistent reduction in TAB at the majority of trial sites. In contrast other cultivars show differing responses across the trial sites eg. KWS Santiago shows a decrease in TAB compared to the trial average at Trial 4, TAB values relatively close to average at Trials 1, 2, 3 and 6 and an increase at Trial 5 while Invicta shows take-all infectivity close to or below average at Trials sites 1, 2, 3, 5 and 6 and an increase at Trial 4.

### Field root system assessment

In two field experiments conducted in different seasons and different fields on the Rothamsted Farm (Hertfordshire, UK) there were no significant differences in crown root number or biomass between the low TAB cultivar Cadenza and high TAB cultivar Hereward as assessed for mature plants during grain development. In 2009/2010 there was a significantly higher average number of seminal roots per plant for Cadenza compared to Hereward (Table 3, Cadenza = 4.60 seminal roots/plant, Hereward = 3.62 seminal roots/plant, P = 0.033). Similar seminal root numbers were recorded between the two field seasons but there were on average between 6–8 additional crown roots in 2014/2015 compared to 2009/2010. As expected there was negligible take-all infection of the 1^st^ wheat root systems in both field experiments but considerable infective *G*. *tritici* inoculum measured in the soil post-harvest in 2014/2015. In 2009/2010 there was minimal take-all inoculum build-up under either wheat cultivar, probably as a result of the dry weather conditions in the spring and early summer as also seen for the rotation field experiment carried out in the same year described above. Under more conducive conditions in 2014/2015 there was a significant reduction in post-harvest take-all infectivity for the low TAB cultivar Cadenza compared to Hereward (Table 3).

**Table 3:** First wheat root phenotyping study for the low TAB cultivar Cadenza compared to the high TAB cultivar Hereward in two field seasons (2009–2010 and 2014–2015).

Field Season	2009/2010				2014/2015				
Cultivar	Square root number of seminal roots per plant (Bt^1^ mean)	Square root number of crown roots per plant (Bt mean)	Logit take-all index (Bt mean)	Logit % roots infected in post-harvest soil core bioassay (Bt mean)	Square root number of seminal roots per plant (Bt mean)	Square root number of crown roots per plant (Bt mean)	Root system dry weight per plant (g)	Logit take-all index (Bt mean)	Logit % roots infected in post-harvest soil core bioassay (Bt mean)
Cadenza	2.222 (4.94)	4.420 (19.54)	−4.31 (0.84)	−4.83 (0.30)	2.145 (4.60)	5.025 (25.25)	0.1983	−3.84 (1.62)	−1.55 (17.20)
Hereward	2.101 (4.41)	4.217 (17.78)	−4.74 (0.37)	−3.87 (1.57)	1.901 (3.62)	5.041 (25.42)	0.1766	−3.93 (1.45)	−0.29 (42.73)
SED	0.061	0.103	0.459	1.183	0.065	0.162	0.015	0.623	0.321
F Probability	0.141	0.141	0.413	0.474	0.033	0.924	0.233	0.899	0.030

^1^Bt = Back-transformed.

### Controlled environment wax layer experiment

To explore root system architecture and biomass in more detail a sand column wax layer experiment was carried out under controlled environment conditions. No differences were found in the overall ability of the low TAB cultivar Cadenza or high TAB cultivar Hereward to penetrate strong layers (Table 4). More roots penetrated the weak wax layer compared to the strong layer for both cultivars. When the numbers of roots penetrating the wax layer were separated into three radial zones, a significant interaction effect of wax strength and cultivar (P < 0.001) was found for the outer most radius between 81 to 102 mm (zone c; Table 4). In this zone 4.46 and 4.38 roots penetrated the weak wax layer for Cadenza and Hereward, respectively. By contrast, in the same outer zone c, no Cadenza roots had penetrated the strong wax layer compared to an average of 2.73 Hereward roots.

**Table 4:** Analysis of variance summary table for number of roots penetrating the wax layer, maximum root depth and radii zones of penetration in a controlled environment wax layer experiment comparing root growth between Cadenza and Hereward in sand columns challenged with either strong or weak paraffin wax layers at 5 cm depth from the surface.

Analysis of variance F Probability					
Variable	Square root number of penetrating roots per plant	Maximum root depth	Radial distance from the seed – square root number of roots penetrating zone per plant^1^		
			A:≤22 mm	B:>22 ≤40 mm	C:>40 ≤51 mm
Cultivar	0.084	0.164	0.717	0.405	<0.001
Strength of wax layer	0.002	0.115	0.057	0.044	<0.001
Interaction	0.240	0.029	0.133	0.568	<0.001

^1^Zone A: diameter 44 mm; zone B: diameter 45–80 mm and zone C: diameter 81–102 mm.

For maximum root depth in the sand column a significant interaction between cultivar and wax strength was also observed (P = 0.029, Table 4). Hereward had significantly deeper roots in the weak compared to the strong wax treatment (28.1 cm and 14.1 cm, respectively) and compared to both of the Cadenza treatments (14.2 cm in weak wax, 17.8 cm in strong wax).

There was a trend for a higher number of seminal roots in Cadenza (P = 0.062, Table 5) which was similar to that observed in root systems collected from field experiments (Table 3). Root dry weight collected from above the wax layer, regardless of strength, was significantly greater in Cadenza compared to Hereward (P = 0.002).

**Table 5:** Controlled environment wax layer root penetration experiment, measures of root architecture at tillering. Average tiller number, number of roots (crown and seminal), dry weight of roots (total, above and below the wax layer), dry weight of shoots and root to shoot ratio.

Cultivar	Number of tillers (n)	Square root of the number of seminal roots per plant (Bt^1^ mean)	Square root of the number of crown roots per plant (Bt^1^ mean)	Dry weight of roots above the wax layer (g)	Dry weight of roots below the wax layer (g)	Total root biomass dry weight (g)	Dry weight of shoots (g)	Root to shoot ratio
Cadenza	5.38	2.313 (5.350)	5.41 (29.268)	0.865	0.524	1.389	2.98	0.467
Hereward	7.75	2.114 (4.469)	5.12 (26.214)	0.566	0.548	1.114	2.25	0.511
SED (df = 33)	0.906	0.093	0.267	0.07	0.099	0.145	0.303	0.065
Effect of cultivar F Probability	0.028	0.062	0.304	0.002	0.813	0.091	0.040	0.518

Data averaged across strong and weak wax layer strengths as there was no significant main effect of wax strength or interaction between wax strength and cultivar.

^1^Bt = back-transformed.

## Discussion

In this study we demonstrate the substantial impact of using first wheat genetics to improve second wheat crop health and thereby enhance grain and straw yields.

Four wheat cultivar rotation trials, started in different field seasons and different fields, revealed that growing a low TAB cultivar in year 1 resulted in less severe take-all root disease and a grain yield advantage of between 0.2–2.4 tonnes/ha in the following second wheat crop in the rotation. This formally tested our hypothesis that 1^st^ wheat cultivar dependent post-harvest differences in TAB are maintained into the following second wheat crop to alter the resulting take-all disease severity and yield. Differences in the amount of take-all disease in the second wheat crop were already apparent at the beginning of stem elongation in the spring and these differences were maintained as the crown root system developed until the summer sampling period during grain filling. Importantly this effect on take-all disease severity and yield was independent of the second wheat cultivar grown i.e. first and second wheat effects were additive. There was some evidence of a main effect of second wheat cultivar on take-all severity suggesting there may be further opportunities to minimise take-all disease by appropriate second wheat cultivar choice. The effect of second wheat cultivar choice on yield was less clear and would need to be explored in further field trials which compared yields in 1^st^ and 2^nd^ wheat situations within the same field trial and season (eg. by including a fallow treatment in year 1).

In two further seasons, sampling selected AHDB winter wheat 1^st^ wheat field trials revealed that variation for the TAB trait was present in elite cultivars, currently grown by commercial farmers, but that there were complex interactions between year, trial site and cultivar. This was particularly noticeable for the percentage of plants infected with take-all in the soil core bioassay. This suggests that multiple low or high TAB mechanisms exist within this set of elite wheat cultivars which are then influenced in different ways by the site specific environmental and agronomic conditions. However, it was still possible to identify a small number of cultivars which show a consistent reduction in TAB across all of their trial sites.

Two of the most consistent cultivars found to provide the low TAB trait were Gallant and Grafton. Both Grafton (parentage = Cordiale × CPBT W97) and Gallant (parentage = (Malacca × Charger) × Xi19) are derived from the low TAB cultivar Cadenza via Cordiale and Xi19 (Pedigree information obtained from AHDB winter wheat pocketbooks https://cereals.ahdb.org.uk/).

To begin to dissect the mechanisms underlying the TAB trait, field and non-field analyses were done to explore specific root traits. Using a controlled environment sand column growth assay, previous studies had linked root penetration through a buried weak or strong wax layer to shallow or deep rooting phenotypes under field conditions^35^. In our study, both Cadenza and Hereward were able to penetrate the strong wax layer to a limited extent. However, neither cultivar showed an increased ability to penetrate the strong wax layer indicating that they are unlikely to possess deep rooting traits in the field. No differences in total root number or total root biomass were identified between Cadenza and Hereward. However, when partitioned into the root mass collected either above or below the wax layer, Cadenza had a greater dry weight above the wax layer regardless of wax strength whereas Hereward was able to maintain a wider lateral root spread under the more challenging strong wax growing conditions. In all other respects, the root spread of the two cultivars were similar. In the field experiments there were also no differences in either crown root number or root biomass between the two cultivars. Collectively, these results do not support the hypothesis that increased root biomass or an unusual root architecture is associated with higher take-all inoculum build-up.

The soil core bioassay method used to phenotype the cultivars for the TAB trait is a measure of the infective ability of take-all inoculum in the soil. As such it could represent changes in both the quantity and quality of inoculum. Comparisons between quantified *G*. *tritici* DNA fungal biomass in the soil and infectivity in the soil bioassay have shown moderate positive correlations between the two methods^32,36^. However, it is possible that other soil chemical, physical and/or biological properties are also occurring to either suppress or promote *G*. *tritici* infectivity. Detailed comparisons between *G*. *tritici* fungal biomass measurements and soil infectivity assays obtained from the same sample would be needed to identify whether there is a direct effect on the quantity of *G*. *tritici* inoculum under different cultivars or if the infective ability of the inoculum has been indirectly altered.

In two related studies, the overall structure of the soil metagenome as well as the genetic and phenotypic diversity of the bacterial species group *Pseudomonas* was examined within year 2 of one of the rotation field experiments^37,38^. The *Pseudomonas* species group was chosen for investigation because first wheat cultivar choice had the largest impact on this species group within the bacterial community. The *Pseudomonas* species group is also of interest because of the reported influence of these bacteria in the build-up of suppressive soils and biological control of take-all disease associated with continuous wheat cropping known as take-all decline^39,40^. Analysis of the genetic and phenotypic diversity of *Pseudomonas fluorescens* populations in year 2 of one of the rotation experiments revealed that, similarly to take-all, there was a substantial effect of first wheat cultivar choice on *Pseudomonas* populations from the wheat rhizosphere^37^. Further investigations by Mehrabi *et al*.^38^ demonstrated that a higher *Pseudomonas fluorescens* species richness was associated with increased take-all disease severity and lower yields. In complementary experimental laboratory microcosm experiments an increase in *Pseudomonas* genotypic diversity impaired the ability of the community to limit *G*. *tritici* fungal growth. Under controlled environment experiments other researchers have also demonstrated that the build-up of take-all suppressive fluorescent *Pseudomonas* populations and metabolite production from these populations are influenced by the wheat cultivar grown^41,42^. Together these results suggest that there may be opportunities to manipulate rhizosphere microbiome development under field conditions for the control of take-all disease using wheat genetics.

The success of the rhizosphere microbiome strategy may be influenced by the composition of the native soil microbiome across different sites. It is already well acknowledged that soil microbial communities differ due to a range of site specific environmental and soil characteristics including pH, nutrient availability, soil moisture, temperature and organic matter content^43^ and previous studies have identified significant soil-type dependent effects on both bulk and rhizosphere soil microbiomes^44–46^. Core differences in the starting soil microbiome and thus the available taxa to be recruited into the plant rhizosphere and endosphere compartments may also help to explain the significant cultivar x site interaction across the AHDB Recommended List trial sites. Despite the significant cultivar x site interaction there were a small number of cultivars which demonstrated a consistent reduction in TAB across all six sites. This raises the possibility that some cultivars can operate more flexibly across a range of soil types, perhaps recruiting different microbial taxa, depending on the starting native community, but which have the same functional effect on take-all inoculum build-up. Future work should focus on the exploring the differences in the soil, rhizosphere and endosphere microbiome of wheat cultivars in relation to the TAB phenotype across different field sites to investigate this potential interaction.

## Materials and Methods

### Wheat cultivar rotation field experiments

Four winter wheat rotation field experiments, each of 2 years duration, were conducted on the Rothamsted Farm (Hertfordshire, UK) during the period 2008–2013. Trials were drilled in the autumn on well-structured flinty, silt clay loam soil (Supplementary Table S5). The trials were arranged as randomised block designs using CycDesigN (VSN International Limited, Hemel Hempstead, UK). In year 1 four replicates of Cadenza (low TAB cultivar) and Hereward (high TAB cultivar) were sown as large plots of 12 m x 78 or 82 m. In Year 2 each of the large plots of Cadenza or Hereward from year 1 were divided into eight 3 m x 9 or 10 m plots and drilled with one of eight commercial winter wheat cultivars (Cordiale, Duxford, Einstein, Gallant, Hereward, Robigus, Solstice and Xi19). Cultivars were chosen to cover a selection of end use quality groups (including bread, biscuit and feed wheats) and 2^nd^ wheat yield performance. Trials were drilled at a seed rate of 350 seeds/m^2^, but were raised (500 seeds/m^2^) for the Cadenza plots in the 1^st^ rotation trial (field trial code 09/R/CS/688) due to poor performance of this seed batch in germination tests. Growth regulator, pesticides, and fertiliser were all applied as necessary according to standard Rothamsted farm practice, except that no seed treatments or fungicides with any reported action against the take-all fungus were used.

After harvest in year 1 each of the year 2 plot positions were marked out using canes. From each of these plot locations five soil cores (5.5 cm diameter by 10 cm deep) were taken in a zig-zag transect, angled underneath the plant rows, for use in a previously published soil core infectivity bioassay^26,47^. The soil cores are turned upside down and inserted into plastic cups but are otherwise undisturbed and contain both bulk and rhizosphere soil as well as root debris from the sampled cultivar. Bait wheat seedlings (cv. Hereward) were grown in the soil cores for 5 weeks before being washed free from soil and assessed for black necrotic take-all lesions.

In year 2 plant samples were taken in both the spring (stem elongation, GS ~ 31) and summer (grain filling, milk and dough development, GS 71-83). Whole plant samples were dug from five 15 cm lengths of row and ten 20 cm lengths of row in the spring and summer, respectively. In the spring the total number of plants per sample and the presence or absence of take-all lesions on each main root axis were recorded^48,49^. In the summer each whole plant root system was visually classified into disease severity categories based on the proportion of the root system showing black necrotic take-all lesions. From this a take-all index was calculated^48,49^.

In the summer the above ground symptoms of disease, premature ripening and stunting of the wheat crop, were also recorded when these symptoms developed. The take-all patch score was assessed visually by eye by estimating the percentage of each plot area showing prematurely ripened stunted plants^49–51^.

At grain maturity (GS = 92) the experiments were combine-harvested and fresh grain weights recorded for a 2 m width cut through the centre of each plot. Grain dry matter was determined by oven-drying 80 g sub-samples of the fresh grain for 16 hrs at 105 °C. In year 2 of the third (2012/R/CS/719) and fourth (13/R/CS/725) rotation field experiments straw yields were also recorded. At maturity a straw cut was made through the centre of each plot. Straw dry matter was determined by oven drying fresh straw samples for 16 hrs at 80 °C. Both grain and straw yields were adjusted to 85% dry matter and scaled to tonnes/ha.

All data was analysed using Genstat (VSNI, Hemel Hempstead, UK)^52^. Percentage disease data was always transformed using the logit transformation, to ensure equal variance. Two-way analysis of variance (ANOVA) procedure was used to analyse the main effect and interaction between year 1 source cultivar and year 2 cultivar. A pooled analysis of the take-all index and yield in year 2 was carried out using the REML procedure in Genstat. Statistically significant effects were supposed when p ≤ 0.05.

### AHDB Recommended List treated winter wheat cultivar field experiments

In total six 1^st^ wheat AHDB Recommended List treated winter wheat cultivar yield trials (full fungicide and plant growth regulator programme) were chosen for TAB phenotyping in 2013/14 and 2014/15 (https://cereals.ahdb.org.uk/varieties/ahdb-recommended-lists.aspx). The six trials were chosen to include a range of soil types, previous cropping histories, sowing dates and geographic locations (Supplementary Table S2). At each trial site three randomised replicated blocks of between 35 to 54 AHDB Recommended List and candidate winter wheat cultivars were grown in incomplete sub-blocking arrangements. At each site plots measured at least 1.1 m x 9 m and seed were drilled to give a target plant population per m^2^ in the spring of 120 for the very early sown trial (Trial 3) and 250 plants per m^2^ for the other five October sown trials. No fungicides with any reported action against take-all disease were used. Yields were recorded at harvest by the AHDB trial contractor and adjusted to 85% dry matter.

Within 1 week of the harvest date of each trial the soil core infectivity bioassay^26,47^ was used to gauge the relative amount of infectious *G*. *tritici* inoculum in the soil.

Initially each trial site was analysed individually with comparisons between cultivars made using the Residual Maximum Likelihood (REML) procedure. Auto-regressive models in both the x and y axis were used to adjust for spatial variation in the distribution of take-all disease when required. Spearman’s rank correlation was then used to assess the association between the percentage of plants and roots infected in the soil core bioassay with grain yield at each site. A pooled analysis of all 6 trials was then conducted with trial site and year included as fixed effects within the REML model to test for interactions between cultivar, trial site and year. Separate residual variances were generated for each trial and the mean cultivar response across all sites formed from weighted combinations from the different trial sites, the weights being inversely proportional to the size of the residual variability.

### Field wheat root system assessment

Two additional 1^st^ wheat field experiments including the cultivars Cadenza and Hereward were conducted on the Rothamsted Farm in the field seasons 2009–2010 (45 cultivars × 4 replicates, Rothamsted Field Great Knott I) and 2014–2015 (6 cultivars x 4 replicates, Rothamsted Field New Zealand). Trials were drilled in the autumn on the 15–16/10/2009 and 20/10/2014 after winter oilseed rape and winter oat crops, respectively. The trials were arranged as incomplete alpha block designs using CycDesigN (VSN International Limited, Hemel Hempstead, UK). During milk development (GS 71-77) plant samples (5 × 20 cm lengths of row per plot) were dug up using a fork from the top 0–10 cm soil horizon in each field trial. The roots were washed free of soil, the tops chopped off and the remaining stem bases and root systems air dried in a polytunnel for 4–5 days before storage at room temperature. Take-all root assessments were carried out as described for the summer plant samples taken from the rotation field experiments above. For the Cadenza and Hereward plots total seminal and crown root counts were carried out on a random sub sample of 10 plants per plot. In the second field experiment (2014–2015) root system dry weights per plot were calculated after drying at 80 °C for 48 hours. Post-harvest the soil core bioassay was used to gauge *G*. *tritici* inoculum build-up beneath Cadenza and Hereward. A square root transformation was carried out on the root count data and the logit transformation used on the disease data to stabilise variance before further analysis. A one-way ANOVA procedure in Genstat (VSNI, Hemel Hempstead, UK) was used to analyse the main effect of cultivar on the measured root and disease variables.

### Controlled environment sand column wax layer experiment

A sand column wax layer method developed for wheat to explore root penetration ability, architecture and biomass^53^ was implemented using a growth tube size of 110 mm internal diameter, 450 mm length and a wax diameter of 102 mm. Two strengths of wax layer were used at concentrations of 30% (strong wax layer) and 5% (weak wax layer) pastillated paraffin wax, which were previously found to have penetrometer resistances of 3.15 and 0.024 MPa, respectively^54^.

The wax layers were installed 50 mm below the surface of plastic growth tubes, filled with sand (Chelford T grade silica sand) and nutrient solution. Growth tubes were arranged in tanks in a fully randomised design of four blocks designed using CycDesigN (VSN International Limited, Hemel Hempstead, UK). One pre-germinated seed of either Cadenza or Hereward was planted per tube. Individual blocks contained 4 tubes with each cultivar being challenged by either a weak or strong wax layer, giving a total of 4 replicates per cultivar at each wax strength.

Root penetration ability, biomass and number were measured after 6 weeks’ growth at day/night 14/8 h, 22/18 °C and 70/80% relative humidity. Light was supplied at 450 μmol m^−2^ s^−1^ photosynthetic photon flux density. At harvest, the total number of roots and the distance of the penetration points from the centre of the wax layer were recorded. The distance was used to calculate the number of roots penetrating within three concentric zones A-C: zone A had diameter of 44 mm, zone B was between diameters of 45 to 80 mm and zone C between 81 to 102 mm. Maximum rooting depth, total root number, growth stage and number of tillers were all recorded. Shoot dry weight and root dry weight both above and below the wax layer were measured after drying for 48 h at 80 °C. All root count data was square root transformed prior to analysis in order to stabilise variance across treatments. All variables were then analysed using a two-way ANOVA procedure to investigate the main effect and interaction between cultivar and wax strength in Genstat (VSNI, Hemel Hempstead, UK)^52^.

### Data availability

The datasets generated during and/or analysed during the current study are available from the corresponding author on reasonable request.

## Electronic supplementary material

Supplementary Information Tables S1-S5

Supplementary Information Table S6
