# Supplementary material for: Exploring the resilience of wheat crops grown in short rotations through minimising the build-up of an important soil-borne fungal pathogen
Source: Sci Rep. 2018 Jun 22;8:9550. doi: 10.1038/s41598-018-25511-8 (PMC6015077; doi:10.1038/s41598-018-25511-8)
Supplement: Supplementary file 1 — Supplementary Information Tables S1-S5 [file 41598_2018_25511_MOESM1_ESM.pdf]

Title

Exploring the resilience of wheat crops grown in short rotations through minimising the build-up of an important soil-borne fungal pathogen

Authors

V.E. McMillan[1], G. Canning[1], J. Moughan[1], R.P. White[2], R.J. Gutteridge[1] and K.E. Hammond-Kosack[1]*

Affiliations

[1] Department of Biointeractions and Crop Protection, Rothamsted Research, Harpenden, Hertfordshire AL5 2JQ, UK

[2] Department of Computational and Analytical Sciences, Rothamsted Research, Harpenden, Herfordshire AL5 2JQ, UK

* Corresponding author: kim.hammond-kosack@rothamsted.ac.uk

Supplementary Information

Table S1 Monthly rainfall (mm) and average maximum temperature (°C) from May to August for the field seasons 2009-2015 on the Rothamsted Farm, Hertfordshire, UK (Data from the electronic Rothamsted archive).

Year	May	June	July	August	Total
Rainfall (mm)					
2009	24.8	68.1	73.4	63.4	229.7
2010	38.5	23.6	31.7	127.8	221.6
2011	23.6	83.0	44.6	81.2	232.4
2012	52.7	166.4	128.4	54.8	402.3
2013	55.9	24.5	47.4	57.5	185.3
2014	82.9	30.4	36.9	113.4	263.6
2015	68.5	26.8	132.6	83.2	311.1
Air Temperature (average t_{max} °C)					Average
2009	17.1	20.0	20.8	22.0	20.0
2010	15.4	20.8	22.8	19.8	19.7
2011	17.4	19.0	19.9	19.9	19.1
2012	16.1	17.6	19.8	21.7	18.8
2013	14.8	18.0	24.5	22.1	19.8
2014	16.5	20.2	23.9	20.0	20.1
2015	15.6	19.5	21.4	20.5	19.2

Table S2 Details of the trial ID, location, sowing date, previous crop and soil type of the six selected AHDB Recommended List winter wheat trials.

Trial Number	Trial ID	Field season	Number of cultivars	County	Nearest Town	Sowing date	Previous crop	Soil Type
1	WW2014SS534T	2013/14	35	Hampshire	Sutton Scotney	11/09/2013	Winter Oilseed Rape	shallow with some chalk
2	WW2014SS527T	2013/14	47	Hampshire	Broughton	24/10/2013	Grain Peas	medium with some chalk
3	WW2014SG526T	2013/14	47	Oxfordshire	Alkerton	08/10/2013	Winter Beans	light sand
4	WW2015SS527T	2014/15	54	Hampshire	Broughton	22/10/2014	Grain Peas	medium with some chalk
5	WW2015WY522T	2014/15	44	Kent	Wye	28/10/2014	Spring Beans	medium
6	WW2015SG526T	2014/15	54	Oxfordshire	Alkerton	22/10/2014	Winter Beans	light sand

Table S3 Correlations between cultivar TAB phenotype and yield in the six sampled AHDB experimental trials.

AHDB winter wheat Recommended List® experiment	Spearman's Rank Correlation	% plants infected with take-all	% roots infected with take-all
Trial 1	Rs	0.183	0.075
	P	0.289	0.667
Trial 2	Rs	0.113	0.034
	P	0.455	0.820
Trial 3	Rs	0.040	0.024
	P	0.804	0.881
Trial 4	Rs	0.030	-0.009
	P	0.833	0.949
Trial 5	Rs	0.064	0.072
	P	0.690	0.652
Trial 6	Rs	0.218	0.274
	P	0.123	0.051

Table S4 Average take-all infectivity of the soil across the two years and 6 sampled AHDB trial sites. The SED and F Probability show the highly significant interaction effect detected between year and trial site.

Year	Trial	Logit % roots infected with take-all (Bt[1] mean)	
2013/2014	1	-0.262	(37.18)
2013/2014	2	-0.490	(27.31)
2013/2014	3	-0.245	(37.97)
2014/2015	4	-1.619	(3.78)
2014/2015	5	-1.776	(2.79)
2014/2015	6	-0.848	(15.51)
SED (Year.Trial)		0.173	
F Probability (Year.Trial)		<0.001	

[1] Bt = Back-transformed

Table S5 Details of the Rothamsted field site, previous cropping history, plot size, sowing date and date harvested of the rotation field experiments.

Rothamsted trial code	Rothamsted Field	Previous cropping history		Plot size (m)	Drilling date	Date harvested
		Preceding year	2 years previous			
**Rotation trial 1**						
Year 1: 2009/R/CS/688	Great Knott 3	Winter oats	Winter wheat	12 x 82	10/10/2008	28/08/2009
Year 2: 2010/R/CS/688	Great Knott 3	Winter wheat	Winter oats	3 x 10	09/10/2009	06/08/2010
**Rotation trial 2**						
Year 1: 2010/R/CS/706	Great Knott 1	Winter rape	Winter wheat	12 x 82	24/09/2009	05/09/2010
Year 2: 2011/R/CS/706	Great Knott 1	Winter wheat	Winter rape	3 x 10	10/10/2010	12/08/2011
**Rotation trial 3**						
Year 1: 2011/R/CS/719	Great Harpenden 2	Winter beans	Winter barley	12 x 82	10/10/2010	31/08/2011
Year 2: 2012/R/CS/719	Great Harpenden 2	Winter wheat	Winter beans	3 x 10	29/09/2011	13/08/2012
**Rotation trial 4**						
Year 1: 2012/R/CS/725	Drapers	Winter beans	Winter wheat	12 x 78	06/10/2011	14/08/2012
Year 2: 2013/R/CS/725	Drapers	Winter wheat	Winter beans	3 x 9	03/10/2012	12/08/2013
